# The quality evaluation system establishment of mesenchymal stromal cells for cell-based therapy products

**DOI:** 10.1186/s13287-020-01696-6

**Published:** 2020-05-13

**Authors:** Yuanyuan Xie, Wei Liu, Shuo Liu, Liudi Wang, Dan Mu, Yi Cui, Yanyan Cui, Bin Wang

**Affiliations:** 1 Clinical Stem Cell Center, The Affiliated Drum Tower Hospital of Nanjing University Medical School, 321 Zhongshan Road, Nanjing, 210000 People’s Republic of China; 2 Department of Radiology, The Affiliated Drum Tower Hospital of Nanjing University Medical School, Nanjing, 210000 People’s Republic of China; 3 Reproductive and Genetic Center of National Research Institute for Family Planning, Beijing, 100081 People’s Republic of China; 4 Department of Cell Biology and Genetics, Chongqing Medical University, Chongqing, 400016 People’s Republic of China

**Keywords:** Cell therapy, Regulations, Policy, Cell quality evaluation, Safety, Mesenchymal stromal cells

## Abstract

**Background:**

Cell-based therapy products are supposed to be the most complex medicine products in the history of human medical care. In this study, we established a safety evaluation system for therapeutic stromal cells based on the existing regulations and current testing techniques to provide general quality requirements for human umbilical cord mesenchymal stromal cell (HUCMSC) therapy product.

**Methods:**

In this system, we comprehensively evaluate the environmental monitoring program, quality control of critical raw materials and reagents, donor screening criteria, cell safety, quality, and biological effects, not only in line with the basic criteria of biological products, but also following the general requirements of drugs.

**Results:**

The qualified HUCMSCs were tested for various clinical researches in our hospital, and no severe adverse reaction was observed in 225 patients during a 1-year follow-up period.

**Conclusion:**

In this study, we establish a systemic quality control and potent assays to guarantee the safety and effectiveness of HUCMSCs based on a minimum set of standards in MSC-based product.

## Background

Over the past couple of years, the mesenchymal stromal cells (MSCs) as one of the cell-based therapy products (CTPs) have attracted great attention due to their broad therapeutic potential. Thus far, MSCs have been used in over 6000 clinical trials worldwide (www.clinicaltrials.gov), and these researches are involved in various diseases and injuries including pulmonary disease [1], cardiac disease [2], neurological disease [3], gliomas [4], graft-versus-host disease (GVHD) [5], and the like. These MSC studies have produced some encouraging achievements, and some of which moved up to clinical practice from preclinical stages, leading to the marketing approval of several CTPs by different national regulatory authorities [6].

In China, the studies of CTPs including MSCs and immune cells have been extensively developed in recent years. The Chinese government has stepped up reforming the regulatory policy regarding CTPs, in alignment with the regulation and policy in the European Union and the USA [7]. In 2015, the Ministry of Health of the People’s Republic of China (MOHC) and the State Food and Drug Administration (SFDA) jointly issued guidelines for stem cell preparation, quality control, and preclinical and clinical trial research management [8–10]. The new guidelines of “Technical Guideline for Research and Evaluation of Cell Products” and “Notice on Strengthening the Preparation and Supervision of Stem Cell Clinical Research” were officially released in 2016 and 2017, respectively [11, 12]. Under the new regulatory policies, CTPs will be categorized into biological drugs and be regulated in light of drug review and monitoring principles; furthermore, the clinical trials of CTPs were only approved to be performed at hospitals authorized by government agencies. Until November 2019, a total of 58 approved clinical trials are carried out in 114 officially authorized hospitals in China.

CTPs are supposed to be the most complex medicine products in the history of human medical care because of their complicated biological characteristics. According to the new regulatory policy, medical institutions authorized to run a clinical trial of CTPs are the main responsible body for the quality of stem cells and clinical research management, namely, authorized institutions (hospitals) must abide by national regulations and guidelines for the safety and efficiency of CTPs, no matter what is produced in commercial companies or in the hospital itself. The quality control of CTPs is very complicated and includes cell facility conditions, donor education and procurement, material requirements, process control and management, toxicological evaluation, biological activity, criteria of storage and release, and others. Our hospital was in the 1^th^list of 30 authorized clinical research hospitals for conducting clinical trials with CTPs in 2015, and now, three approved clinical trials of CTPs are running in our hospitals. We have established Good Manufacturing Practice (GMP) cell facility in our hospital and produced human umbilical cord MSCs (HUCMSCs) for approved clinical trials.

To ensure the quality and effectiveness of HUCMSCs in clinical trials, we established a safety evaluation system for therapeutic MSCs based on the existing regulations and current testing techniques [13, 14], which provide general quality requirements for MSC therapy product. The main reference documents include but not limited to the guidelines for cell bank characterization in the Chinese Pharmacopoeia (part III, 2015 version) [15] and the International Society for Stem Cell Research (ISSCR) [13]. To ensure the quality consistency of the final products of each batch, we established the Master Cell Bank (MCB) and Working Cell Bank (WCB) models. In this system, we comprehensively evaluate cell safety, quality, and biological effects, not only in line with the basic criteria of biological products, but also following the general requirements of drugs. We hope our system could contribute to establishing the evaluation framework of CTPs for advancing the steadfast development of stem cell-based therapies.

## Materials and methods

This study was approved by the Research Ethics Board of Nanjing Drum Tower Hospital. Written consent was obtained from the donors and recipients.

### Environmental monitoring program

HUCMSCs are manufactured in clean environments in accordance with the requirements of current GMP (cGMP) [16]. Complying with cGMP means the production of therapeutic MSCs requires careful identification and control of all the phases of production. Therefore, in any MSC bank, whether for research or therapy, it is necessary to design and implement an environmental monitoring program to minimize the introduction, generation, and retention of particles and microorganisms in the final product.

### Quality control of critical raw materials and reagents

The use of substandard raw materials and reagents may lead to the contamination of HUCMSCs; therefore, quality control for critical raw materials and reagents is required. The critical raw materials and reagents used in cell culture include fetal bovine serum (FBS) (Gibco, USA), TrypLE (Gibco, USA), phosphate-buffered saline (PBS) (Gibco, USA), and culture flask (Corning, USA). First, we should ensure that materials and reagents used for cell therapy are purchased from qualified manufacturers that guarantee their GMP compliance production and then certificates should be obtained too. As stated in the regulations, GMP-compliant FBS can be used for the preparation of therapeutic grade stem cells, but the serum must be free of bovine-specific viruses including bovine viral diarrhea virus (BVDV), bovine parainfluenza virus (BPIV), bovine parvovirus (BPV), bovine adenovirus (BAV), and reovirus (REO). Immunofluorescence was performed to detect these bovine-specific viruses as shown in Supplementary Table 1 according to the previous method [17]. Briefly bovine turbinate (BT) cells and Vero cells were cultured with the test bovine serum for detecting BVDV, BPIV, BPV, and BAV and for detecting REO, respectively. The cultured BT cells and Vero cells were stained with corresponding fluorescent antibodies as listed in Supplementary Table 1. The bovine serum was released to culture the clinical HUCMSCs only when all the tests of viruses were negative. The digestive TrypLE solution for HUCMSCs was a recombinant cellular digestive enzyme produced from the bacterial expression system, without a contamination risk of animal- or human-specific viruses.

### Donor screening criteria

The cell-based therapy has the potential to transmit infectious diseases. The age, delivery mode, and health status of the donor can also affect the quality and function of HUCMSCs [18]. Thus, a strict donor screening needs to be performed before sample collection, including detailed medical history, physical examination, and transmissible disease testing. As for the detection of transmissible diseases, diseases including without limitation hepatitis B virus (HBV), hepatitis C virus (HCV), human immunodeficiency virus (HIV I and II), syphilis, cytomegalovirus (CMV), and Epstein-Barr virus (EBV) were detected using ELISA or nucleic acid detection method. In order to exclude the window period of these viral infections, we will have other serological tests of infectious diseases for the donor 3 months after UC donation [19].

### Isolation and culture of HUCMSCs

With the consent, a 15-cm long fresh UC was obtained from a full-term delivery donor, and stored in sterile PBS added with 100 IU/mL penicillin and 100 μg/mL streptomycin (PS, Gibco, USA), and then immediately transferred to the GMP cell facility in a shipment box at 2~6 °C. The primary HUCMSCs were isolated using a tissue explant method [20]. The cells were digested and transferred into new culture bottles for further expansion when well-developed colonies of fibroblast-like cells appeared. The cells of the second and fourth generations were harvested and cryopreserved in liquid nitrogen tanks as MCB and WCB cells, respectively. Before being cryopreserved and released, the samples of supernatant and cells were sent to the quality control lab for in-process and release testing.

### Bacteria and fungi contamination testing

Microbial contamination of cell products can lead to a fatal outcome in the clinic [21]. Bacteria or fungi are common microbial contamination during cell culturing and can occur at all stages of the sample collection, cell cultivation, storage, and transport process. The 2-mL supernatant samples were inoculated in BacT/ALERT Aerobic (Merieux, France) and Anaerobic Culture Bottle (Merieux, France) with sterile syringes and incubated at 37 °C for 7 days. In this BacT/ALERT system, aerobic and anaerobic bacteria, as well as fungal contamination, could be detected and the testing period was shortened down to 7 days through a continuous reading of carbon dioxide [21, 22]. In addition, Gram staining was performed before the release of the final cell product as a supplementary experiment for sterility assay. All results of sterility assay must be negative; otherwise, the cells were judged as unqualified.

### Endotoxin assay

Endotoxin is the cell wall component of various gram-negative bacteria, which is released after the lysis of the bacteria, also known as “pyrogen.” The presence of endotoxin is also evidence of gram-positive bacterial infection. The contamination of endotoxin can cause adverse reactions in patients such as fever, irreversible septic shock, disseminated intravascular coagulation, and even death. Endotoxin was detected by Gel Clot Endotoxin Assay Kit (GenScript, USA) based on the manufacturer’s protocols. The endotoxin level of samples below the detection limit (< 0.5 EU/mL) is considered acceptable.

### Mycoplasma assay

Mycoplasma is the smallest and simplest self-replicating prokaryote, and more than 200 species of mycoplasma have been described so far. There are seven mycoplasmas accounted for 98% of mycoplasma contamination during cell culture, including *M. hyorhinis*, *M. arginini*, *M. orale*, *M. fermentans*, *M. salivarium*, *M. hominis*, and *Acholeplasma laidawii* [23]. A variety of methods have been developed to test mycoplasma contamination. The microbiological culture and DNA fluorescence staining are the classic methods recommended by the Chinese Pharmacopoeia, but are relatively time-consuming, not appropriate for quick release inspection before the clinical infusion of HUCMSCs. The PCR method is an optional mycoplasma testing method because it is very sensitive, specific, and time-saving. The One-Step Quickcolor Mycoplasma Test Kit (CLARK Bioscience, USA) was used according to the manufacturer’s instruction.

### Cell counts and viability

The cell numbers were determined using an automatic cell counter (Nexcelom, Cellometer Mini, USA), and the trypan blue exclusion method was used for cell viability detection. In addition, the fourth passage cells were harvested for cell proliferation, apoptosis, growth curve, and cell cycle assays as a complementary experiment to decipher the viability of cells. The 5-ethynyl-2′-deoxyuridine (EDU, RiboBio Co., China) and Cell Counting Kit (Beyotime, China) were performed according to the manufacturer’s instruction, then the proliferation rate and growth curve were calculated or drawn, respectively. The apoptosis assay was performed with the Annexin V-FITC Apoptosis Detection Kit (Vazyme, China). The BD Cycletest™ Plus DNA Kit (BD, USA) was used to determine the cell cycle. Before releasing the final cell products, the cell count and viability assay also were performed and the viability must be over 85%. A tumor cell line (murine melanoma B16F10 cell) was cultured in an independent incubator as a positive cell control in all the above experiments because of its rapid and stable growth rate. In cell viability and apoptosis assays, a dose of 800 μM H_2_O_2_ was added to HUCMSC culture medium to induce cell apoptosis as positive controls to ensure the reliability of the experiments.

### Cell identification

The definitive identification of cells is the first problem that needs to be solved in cell therapy products. The settings of the cell identity standard facilitate the exchange of data among researchers and distinguish any admixed cell population. HUCMSCs have three minimal definition criteria including adhesion to plastic, specific surface marker expressions (CD105, CD73, CD90, positive cells ≥ 95%, CD14 or CD11b, CD34, CD45, CD79a or CD19, and HLA-DR-positive cells ≤ 2%), and multilineage differentiation potentials of adipogenesis, osteogenesis, and chondrogenesis according to the guidelines from the Mesenchymal and Tissue Stem Cell Committee of the International Society for Cellular Therapy (ISCT) [24]. For surface marker expression assay, approximately 1 × 10^6^ cells at the fourth passage were harvested and resuspended in 100 μL PBS, following being stained with the following monoclonal antibodies labeled with either fluroisothiocyanate (FITC) or phycoerythrin (PE): CD34, CD11b, CD45, CD19, CD73, CD105, CD90, and HLA-DR (BD, USA). After incubation in the dark for 30 min at room temperature, cells were washed three times by 1× PBS and resuspended in washing buffer for flow cytometry analysis (BD FACSAria™, USA). The analysis data was analyzed with the FACS software. In regard to multilineage differentiation, HUCMSCs at the fourth passage were harvested and replated at a density of 1 × 10^4^ cells/well in a 24-well culture plate. When the cells reached 50~70% confluency, adipogenic and osteogenic media (Gibco, USA) were replaced to induce adipogenesis and osteogenesis, respectively. After 21 days, cells were fixed in 4% formaldehyde and stained with Oil red O (Sigma-Aldrich, USA) or Alizarin Red S (Sigma-Aldrich, USA) to evaluate the adipogenic or osteogenic differentiations, respectively. In addition, 2 × 10^5^ cells at the fourth passage were centrifuged for 5 min at 1200 rpm/min in a tube, and the chondrogenic medium was (Gibco, USA) added in the pellet after removal of the supernatant to evaluate the chondrogenic differentiation of HUCMSCs. After 21 days, the pellet was fixed in 4% formaldehyde, dehydrated through serial ethanol dilutions, and embedded in optimal cutting temperature compound (OCT). Blocks were cut into 5-mm-thick sections and stained with Alcian Blue (Sigma-Aldrich, USA).

### Safety evaluations

Studies have shown that MSCs have 4% possibility of chromosomal abnormality during the in vitro culture, and the tumorigenicity of stem cells is also a major safety concern in clinical applications [25]. The karyotype and tumorigenicity assay in immunodeficient mice were performed to validate the genetic stability and tumorigenicity potential of HUCMSCs; Giemsa banding technique was used for karyotype analysis. Briefly, the cells at the fourth passage were treated with colchicine solution and incubated at 37 °C for 30 min before harvesting. Then the cells were fixed and spread with standard procedures. Twenty metaphases were analyzed. Chromosomal abnormalities include the number abnormalities and morphological distortion. In regard to tumorigenicity, male mice with severe combined immune deficiency (SCID) were injected subcutaneously with 1 × 10^7^ HUCMSCs or human embryonic stem cells (HESCs) as positive control and monitored once a week to record the formation of tumors, lasting for 4 months. The mice were sacrificed after anesthetized excessively, and major organs were harvested for hematoxylin-eosin (H&E) staining.

### Immunomodulation assay

Accumulating evidence has shown that the immunomodulatory function of MSCs is the basis for curing various diseases such as systemic lupus erythematous (SLE) and osteoarthritis, which is recommended as a potency release criterion for advanced phase clinical trials by the ISCT [26]. In our evaluation system, the immunomodulatory effects of HUCMSCs on Th1, Th17, and the regulatory T cells (Tregs) were assayed by co-culturing HUCMSCs with human peripheral blood mononuclear cells (PBMCs). The method was briefly described below. HUCMSCs were plated into 6-well plates (1 × 10^5^/well) and were incubated for 24 h with Roswell Park Memorial Institute (RMPI) 1640 complete medium. Human PBMCs were prepared from leukapheresis packs by centrifugation on a Ficoll Hypaque density gradient (AXIS-SHIELD, NOR). PBMCs were then plated into 6-well plates in the presence or absence of HUCMSCs (HUCMSCs/PBMCs ratio, 1:10). For Treg population assay, rhIL-2 (5 ng/mL) was needed to be added to the culture medium. After co-culturing for 3 days, T cells were collected and stimulated for 5 h with 1× compound stimulant of cocktail (Invitrogen, USA). Cells were first stained with CD3-Percp and CD8-APC (BD, USA) and incubated for 15 min at room temperature. Then, cells were fixed and permeabilized using the Cell Fixation & Permeabilization kit (FMS, China) according to the manufacturer’s instructions. After washing, we performed intracellular staining for IFN-γ-FITC and IL-17A-PE detection. For the detection of Tregs, non-adherent cells were harvested and evaluated according to the manufacturer’s instructions (eBioscience, USA). Cells were analyzed through flow cytometry (BD FACSAria™, USA), and data were analyzed with the FACS software.

### Statistical analysis

Data were expressed by mean ± standard deviation (SD) from triplicate or more experiments. The statistical analysis of data was performed using the GraphPad Prism 6 software (GraphPad Software, USA). Comparisons of quantitative data were performed with one-way ANOVA (S-NK). *p* value < 0.05 was supposed to have a statistical sense.

### Adverse effects of HUCMSCs in clinical trails

The “qualified HUCMSCs” after systemic quality evaluation have been applied to clinical trials executed in our hospital including chronic ischemic cardiomyopathy (NCT02635464), recurrent uterine adhesion (NCT03592849), type 1 diabetes (NCT02763423), and premature ovarian failure (NCT02644447). The adverse effects in recipients were monitored to further validate the safety of HUCMSCs in vivo.

## Results

### Environmental monitoring program meeting the standard requirements

All environmental parameters of the GMP laboratories such as particle and microbiological monitorings should be within normal limits and are strictly controllable prior to any operation. The microbiological quality of all sampled points was evaluated by counting the colony-forming units (cfu) in the dish in each cell culture facility including the preparation and operation room unit. A total of 9 air point samples and 5 surface point samples were harvested for each unit, and one hundred and sixty-eight points were assessed each unit per year. The alert and action limits of surface and air are 0.2 cfu/30 min/dish and 4 cfu/30 min/dish, respectively. As shown in Table 1, the levels of contamination were always in standard permission scope. The particles in the GMP cell culture facility were monitored once a year by laser dust particle counter/BCJ-1 detection whose measuring range/accuracy was 0.3~5.0 μm. Five points in each cleanroom were sampled from the air in each particle monitoring. The alert and action limits of class 1000 cleanliness level in the GMP environment air need to meet three criteria simultaneously: 0.3 μm ≤ 309 particles/2.83 L min, 0.5 μm ≤ 100 particles/2.83 L min, and 1.0 μm ≤ 24 particles/2.83 L min. The particle monitoring data shown in Table 2 were in the permission scope.

**Table 1:** The microbiological monitoring data of sampled points in the GMP cell facility in the year 2019

Sampling points (cfu/30 min/dish)	January	February	March	April	May	June	July	August	September	October	November	December
Surface (5 points)	0.0 ± 0.0	0.0 ± 0.0	0.0 ± 0.0	0.1 ± 0.3	0.0 ± 0.0	0.1 ± 0.3	0.0 ± 0.0	0.1 ± 0.3	0.1 ± 0.3	0.1 ± 0.3	0.2 ± 0.4	0.0 ± 0.0
Air (9 points)	0.0 ± 0.0	0.3 ± 0.5	1.4 ± 0.5	0.3 ± 05	0.1 ± 0.2	0.0 ± 0.0	0.2 ± 0.4	1.0 ± 0.6	0.4 ± 0.5	0.6 ± 0.6	0.1 ± 0.3	0.0 ± 0.0

**Table 2:** The particle monitoring data of the GMP cell facility in the year 2019

Sampling points	Particles/2.83 L min		
	0.3 μm	0.5 μm	1.0 μm
1	6.0	4.0	1.0
2	5.0	5.0	3.0
3	5.0	3.0	0.0
4	5.0	5.0	3.0
5	10.0	8.0	1.0
Mean	6.2	5.0	1.6
SD	1.9	1.7	1.2

*SD* standard deviation

### Donor screening criteria

UC donors are healthy full-term puerperas aged under 35 years, without family hereditary disease history, malignant tumor, and transmissible diseases. We screened a list of communicable diseases for the donor as shown in Table 3 a week before the UC collection (first time) and 3 months later after UC collection (second time). All infectious diseases must be proved to be negative in twice screenings, which is considered as qualified in communicable disease screening.

**Table 3:** The screening of communicable disease viruses for donors

	HBsAg	Anti-HBs	HBeAg	Anti-HBe	HBcAb	HCV Ab	HIV Ab	TP Ab	HBV DNA (IU/mL)	HCV DNA (IU/mL)	HIV DNA (IU/mL)	TP DNA (IU/mL)	CMV DNA (IU/mL)	EBV DNA (IU/mL)
First	–	+/−	–	–	–	–	–	–	< 100	< 100	< 100	< 100	< 100	< 100
Second	–	+/−	–	–	–	–	–	–	< 100	< 100	< 100	< 100	< 100	< 100

*HBsAg* hepatitis B surface antigen, *anti-HBs* antiboby to HBsAg, *HBeAg* hepatitis B e antigen, *anti-HBe* antibody to HBeAg, *anti-HBc* antibody to hepatitis B core antigen

### Surface marker expressions of HUCMSCs

**Fig. 1:**
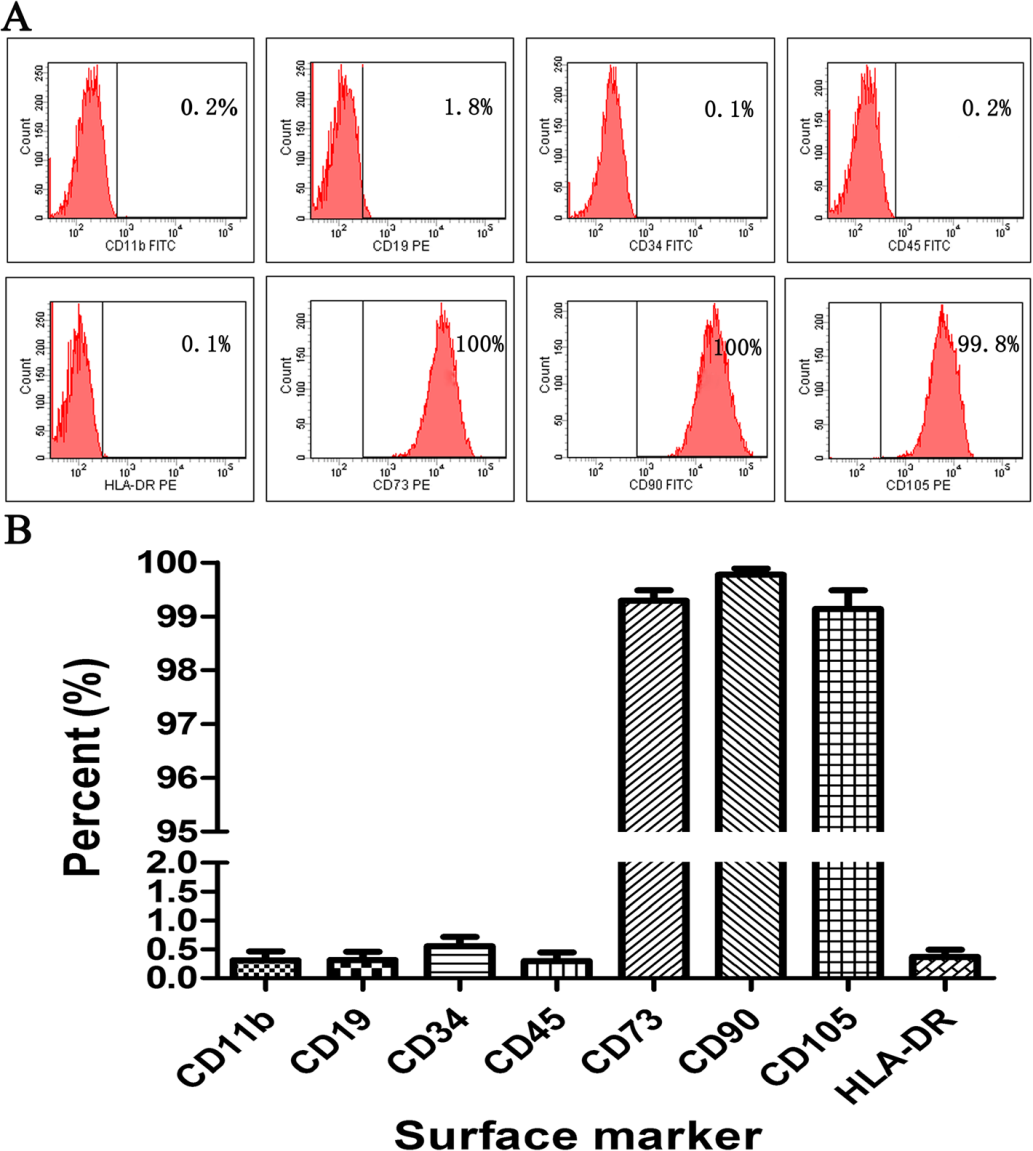
Characterization of HUCMSCs. **a** HUCMSCs negatively expressed CD11b, CD19, CD34, CD45, and HLA-DR but positively expressed CD73, CD90, and CD105 by flow cytometry analysis. **b** Statistical analysis: the percentages of each positively and negatively expressed surface marker HUCMSCs are greater than 95% and less than 2%, respectively

The flow cytometry analysis showed that HUCMSCs positively expressed surface markers of CD73, CD90, and CD105 and negatively expressed surface markers of CD19, CD34, CD11b, CD 45, and HLA-DR (Fig. 1). The percentages of each positively and negatively expressed surface marker HUCMSCs should be greater than 95% and less than 2%, respectively.

### The viability and proliferation of HUCMSCs

**Fig. 2:**
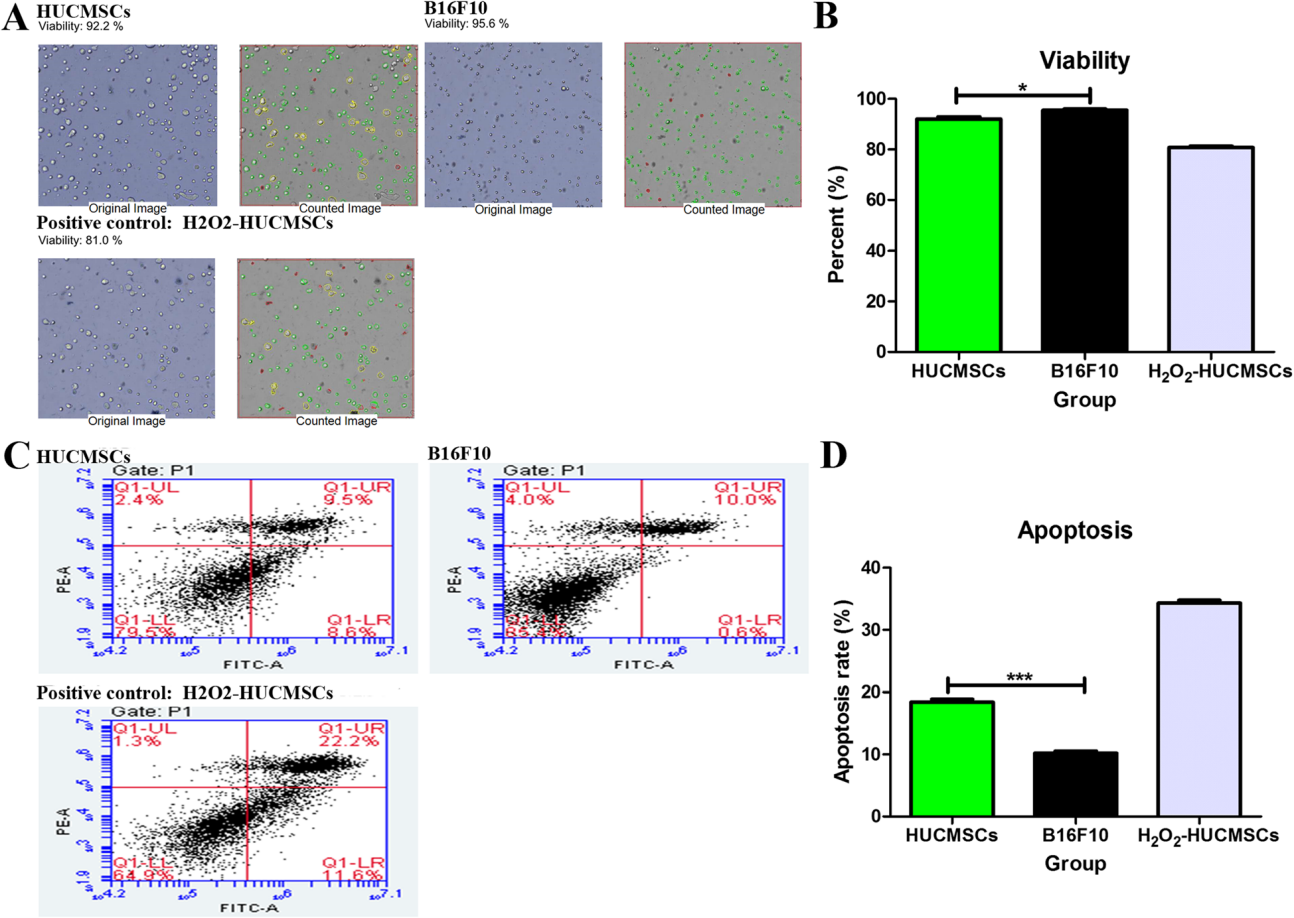
The viability and apoptosis of HUCMSCs and B16F10 cells. **a** The trypan blue exclusion data for viability. **b** B16F10 had a higher survival rate than HUCMSCs with a statistical significance. **c** Annexin V-FITC detection results for apoptosis. **d** HUCMSCs had about 18% apoptosis rate, higher than that of B16F10 (*p < 0.05; ***p < 0.001)

The HUCMSCs as a cell therapy product should have good viability and growth potential to ensure their therapeutic effects in vivo. In our practice, the trypan blue exclusion method showed that the final HUCMSC product had a well viability over 90%, which was much better than the national recommended standards for stem cell viability (> 85%) (Fig. 2a) [27]. As a positive control and reference standard, B16F10 had a higher survival rate than HUCMSCs with a statistical significance (Fig. 2b). In addition, HUCMSCs generally were suspended in saline with or without 5% human serum albumin (HSA) for local or intravenous infusion into patients in clinical trials. Sometimes the cell infusion could not be performed in time because of a transportation delay or other issues. We also assayed the viability of HUCMSCs suspended in saline alone or saline with 5% HSA at 4 °C at different time points. The results showed HUCMSCs suspended in saline with 5% HSA (1 × 10^6^ cells/mL) had a markedly higher viability than HUCMSCs suspended in saline alone at all indicated times (Supplementary Figure 1). Thus, we recommend that the cell infusion should be completed as soon as possible once the cell product is released. Flow cytometry analysis showed that HUCMSCs had about 18% apoptosis rate, higher than that of B16F10 (Fig. 2c, d). H_2_O_2_ (800 μM) treatment induced a significant viability decrease and apoptosis increase in HUCMSCs, indicating viability and apoptosis assays were reliable.

**Fig. 3:**
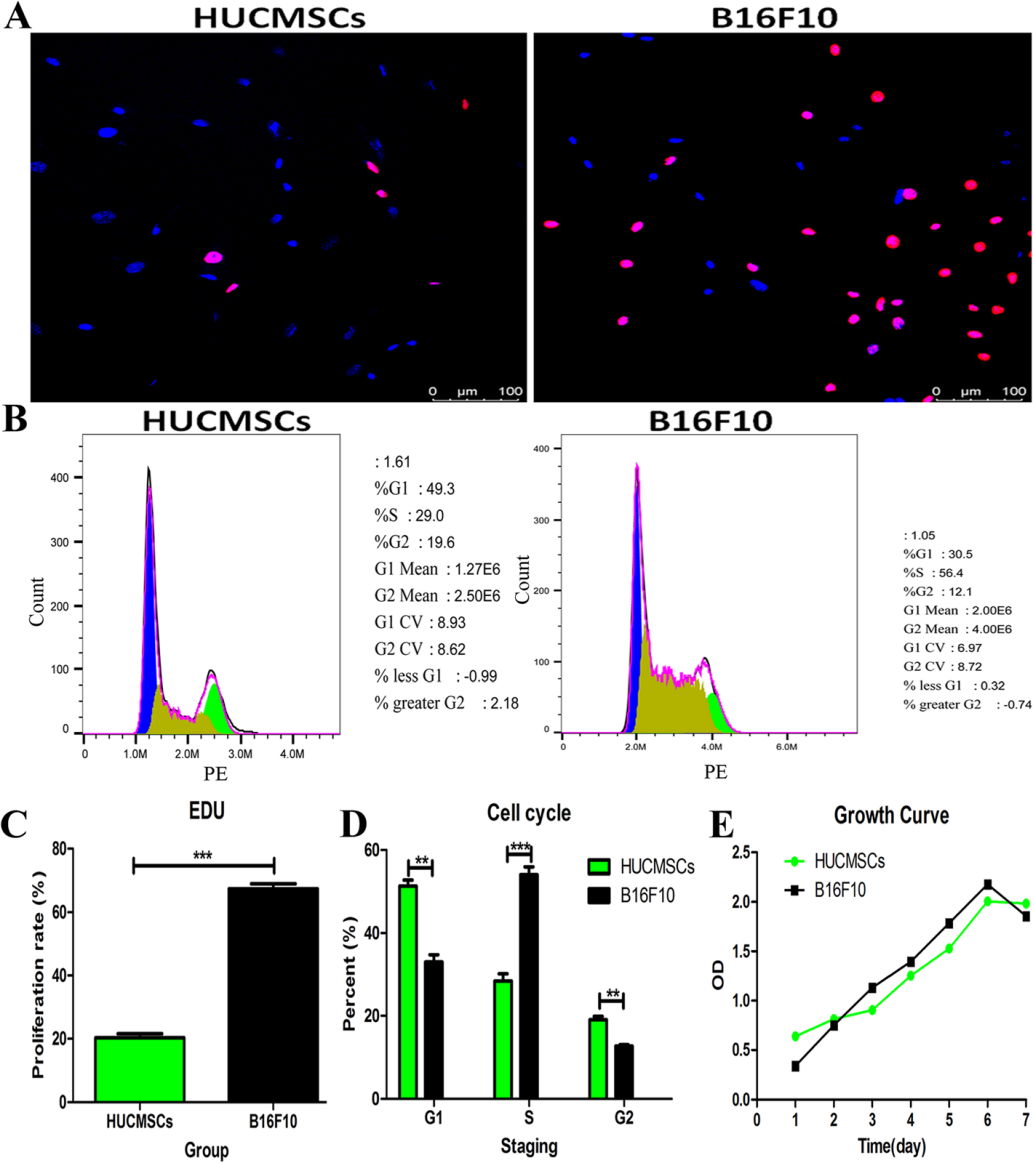
The growth potential of HUCMSCs and B16F10 cells. **a** The red nuclei cells were identified as proliferating cells in EDU immunostaining for proliferation rate. **b** FCM analysis data for cell cycle. **c** HUCMSCS had a much lower proliferation rate than that of tumor cell line B16F10. **d** The percentage of B16F10 in the S phase was statistically higher, but in G1 and G2 phases were both lower than that of HUCMSCs. **e** HUCMCs had a “S” growth curve, but the growth of HUCMSCs was inferior to B16F10 (**p < 0.01; ***p < 0.001)

To evaluate the growth potential of HUCMSCs, the proliferation, cell cycle, and growth curve were assayed by EDU immunostaining, FCM analysis, and CCK-8 assay, respectively (Fig. 3). In EDU immunostaining, the red nuclei cells were identified as proliferating cells (Fig. 3a), and HUCMSCs had a much lower proliferation rate than that of tumor cell line B16F10 (Fig. 3c). Cell cycle analysis showed that the G1, S, and G2 phases in HUCMSCs accounted for about 50%, 30%, and 20%, respectively (Fig. 3b), but the S phase of B16F10 was statistically higher compared with HUCMSCs (*p* < 0.001, Fig. 3d), indicating HUCMSCs had a much lower growth potential than B16F10. Similarly, the growth curve showed HUCMSCs and B16F10 both had a well growth, but HUCMSCs was inferior to B16F10 (Fig. 3e).

### The tumorigenicity of HUCMSCs

**Fig. 4:**
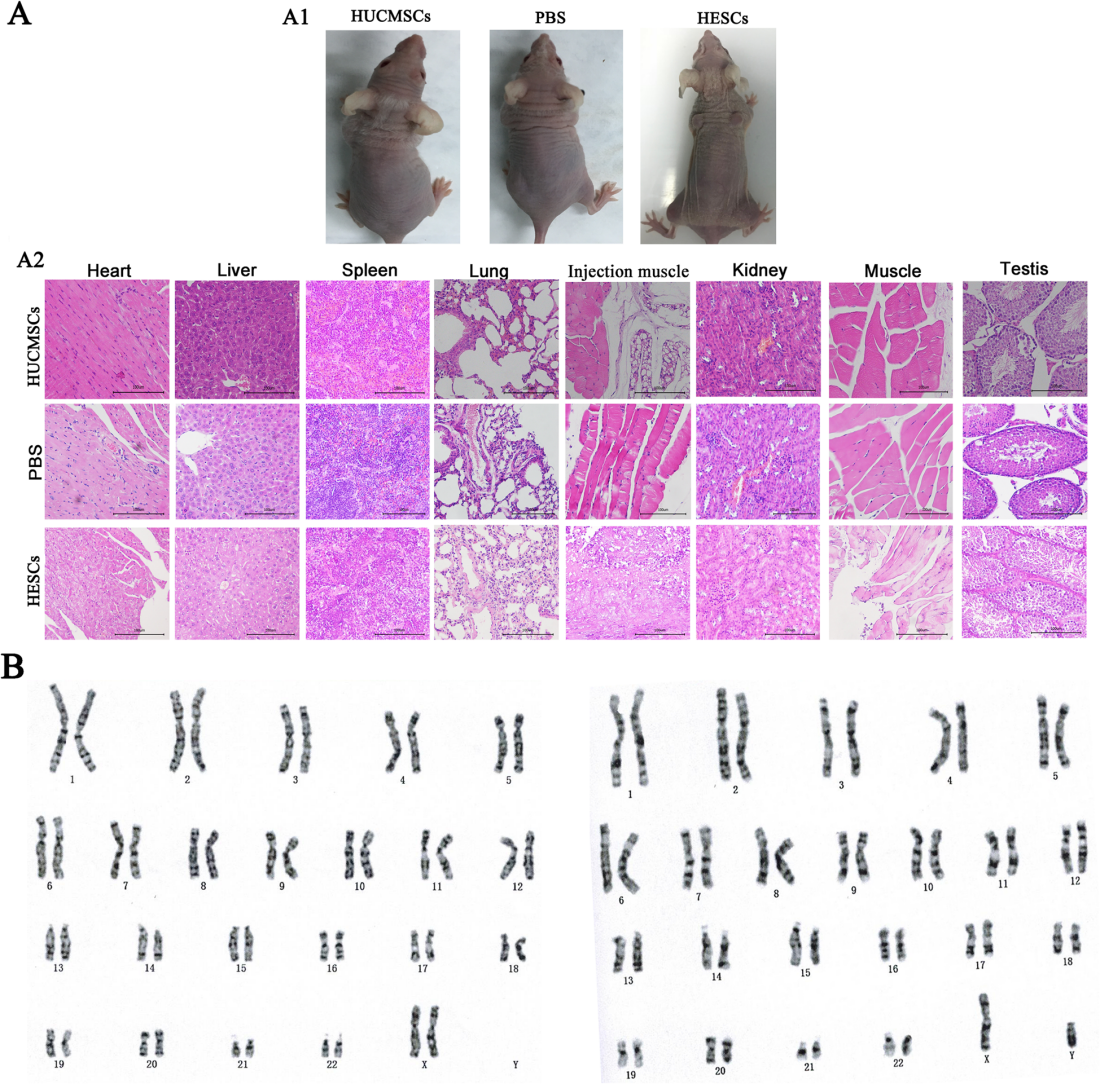
The tumorigenicity of HUCMSCs in SCID mice. **a** The tumor formation was observed in the SCID mice injected with HESCs, but there was no tumor formation observed in any SCID mice in the HUCMSCs group or PBS group and H&E staining showed no infiltration of tumor cells at the cell injection site and main organs. **b** The karyotype of HUCMSCs is normal with 46 chromosomes (XX/XY)

The risk of tumorigenicity of transplanted MSCs is a major safety concern. The HUCMSCs were subcutaneously injected into double-neck sides of SCID mice to investigate tumor formation during a long-term observation period (4 months). As Fig. 4 (A1) showed, in positive control SCID mice injected with HESCs, the tumor formation was observed soon (within about 4 weeks after transplantation). But there was no tumor formation observed in any SCID mice in the HUCMSCs group, and H&E staining showed no infiltration of tumor cells at the cell injection site and main organs including sexual organs, heart, spleen, liver, lung, kidney, muscle, skin, uterus, ovary, and testis tissues (Fig. 4 (A2)).

### The genetic stability of HUCMSCs

The qualified HUCMSCs should have a stable genetic stability, and karyotype analysis was performed to investigate the genetic stability of HUCMSCs. The normal karyotype has 46 chromosomes (XX/XY), without any abnormality in number, morphology, length, size, centromere position, deletion, reduplication, inversion, translocation, insertion, and Ring chromosome in karyotype analysis (Fig. 4b).

### The differentiation potential assay of HUCMSCs

**Fig. 5:**
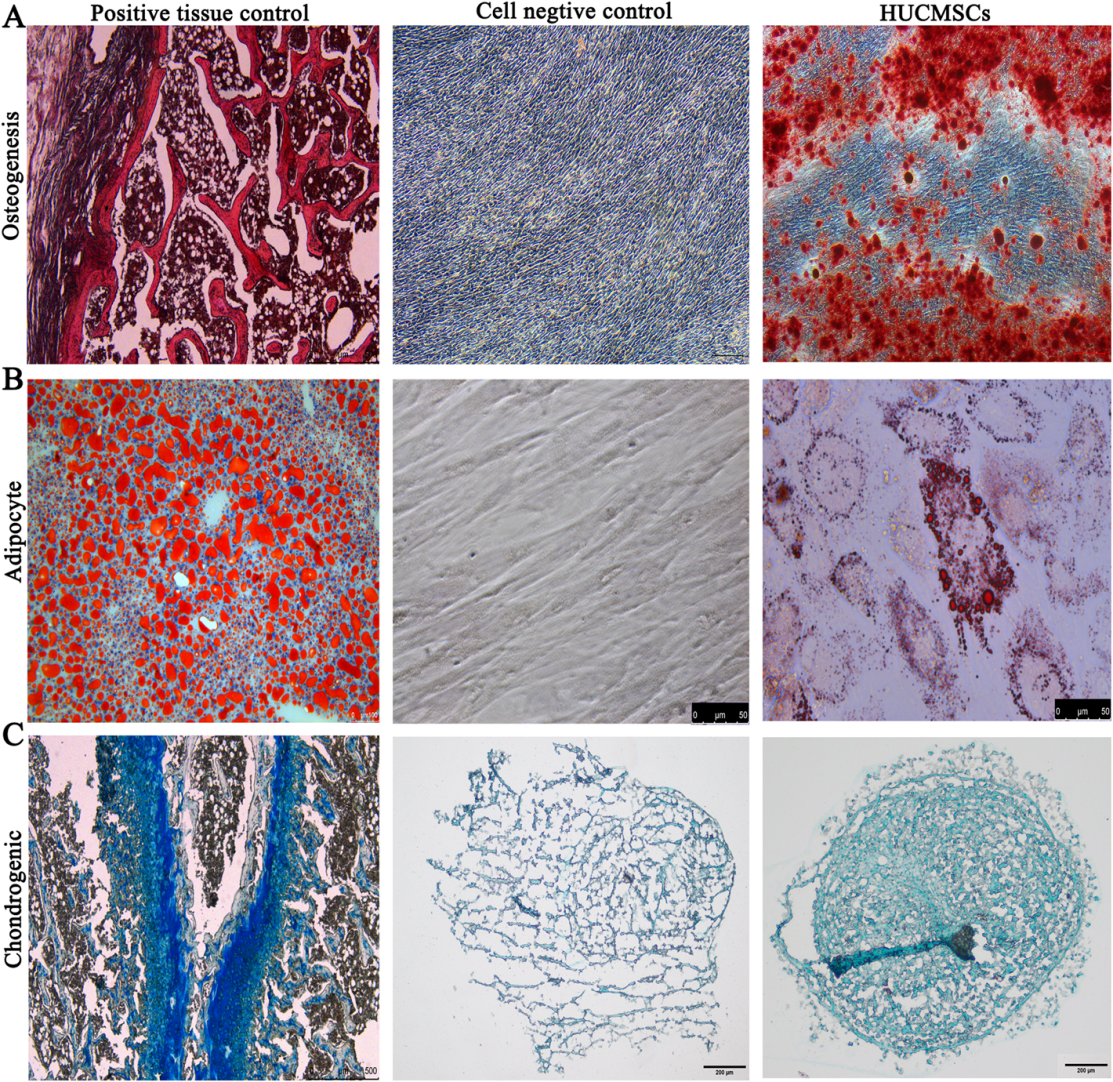
Positive controls: adipose tissue staining for adipocyte differentiation, bone tissue of mouse knee staining for osteocyte differentiation, and cartilage tissue of mouse knee for chondrocyte differentiation. Alizarin Red S staining, Oil red O staining, and Alcian Blue staining showed HUCMSCs were induced into osteogenic, adipogenic, and chondrogenic cells, respectively

The multiple lineage differentiation potentials are an important characteristic of MSCs. As shown in Fig. 5, we established an effective evaluation system to assay the differentiation potentials of HUCMSCs including osteocytes, chondrocytes, and adipocytes. For each differentiation assay, we established a positive control such as adipose tissue staining for adipocyte differentiation, bone tissue of mouse knee staining for osteocyte differentiation, and cartilage tissue of mouse knee for chondrocyte differentiation. HUCMSCs were judged to own osteogenic, adipogenic, and chondrocytic differentiation capabilities through comparing the tested HUCMSC sample staining with the negative and positive stainings.

### Immunomodulation effects of HUCMSCs

**Fig. 6:**
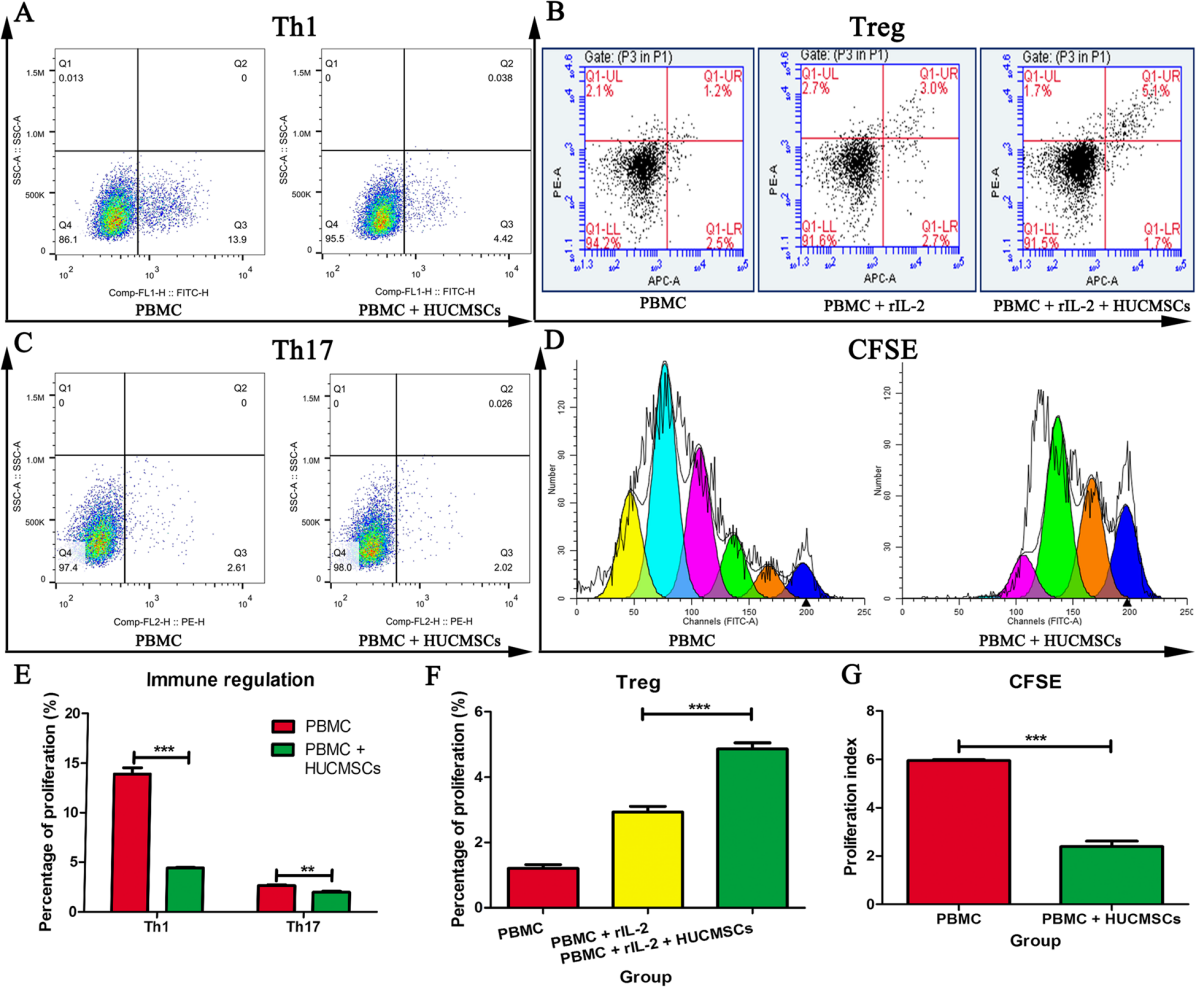
The immunomodulatory effects of HUCMSCs were assayed by PBMCs co-culturing with HUCMSCs. HUCMSCs suppressed the activation and differentiations of CD4+ T cells into Th1 and Th17 subpopulations (**a**, **c**, **e***p* < 0.001), promoted the maturation of Treg subpopulation in PBMCs induced by IL-2 (**b**, **f***p* < 0.001), and inhibited CFSE-labeled PBMC proliferation (**d**, **g***p* < 0.001)

It is well known that MSCs function as immune mediators in curing various immune diseases by secreting immune mediators or direct interaction with immune cells in recipients. We assayed T cell subpopulation of PBMCs co-culturing with HUCMSCs to evaluate the immunomodulatory effects of HUCMSCs. HUCMSCs remarkedly inhibited CFSE-labeled PBMC proliferation by co-culturing and suppressed the activation and differentiations of CD4+ T cells into Th1 and Th17 subpopulations (*p* < 0.001). In contrast, HUCMSCs significantly promoted the maturation of Treg subpopulation in PBMCs induced by IL-2 (*p* < 0.001) (Fig. 6).

### Release testing of HUCMCs before clinical application

Before clinic use, HUCMSCs underwent the fast and relative brief release testing as last safeguard measure, to ensure the safety of recipients. The release assays included bacterial, fungi, endotoxin, mycoplasma, cell amount and viability, and BSA residual level. All results were should be acquired before the distribution of HUCMSCs and were full compliance with the release criteria (Table 4). We can also know that most of the cells are in G1 and S phases (see Fig. 3).

**Table 4:** Summary of quality controls performed on HUCMSCs and compliance with the release criteria

Test	Method	Release criteria	Compliance
Sterility (aerobic, anaerobic, fungal)	BacT/ALERT, Gram stain	No growth at 7 days	225/225
Endotoxin	Gel Clot	< 0.5 EU/mL	225/225
Mycoplasma	PCR	Negative	225/225
Viability	Trypan blue dye exclusion	≥ 85%	225/225
BSA	ELISA	No detect	225/225

### The qualified HUCMSCs were safe in clinical applications

Our center has established a complete set of safety evaluation and quality control systems for stem cell preparation, and then, qualified cells were provided for clinical research. The clinical trials on stem cells conducted by the Nanjing Drum Tower Hospital and the basic situation of patients are summarized in Table 5. Adverse events reported in patients after HUCMSC graft infusion were recorded in Table 6.

**Table 5:** Characteristics of HUCMSC infusion patient

Patients and diseases	Number	Percentage	Median age (years)
Men	65	71.1	
Women	160	28.9	
Premature ovarian failure (POF)	151	67.1	37
Coronary artery disease (CAD)	40	17.8	65
Diabetic erectile dysfunction	26	11.5	46
Type 1 diabetes mellitus	4	1.8	21
Nasal septum injury	2	0.9	24
Liver failure	2	0.9	70
Total	225	100

**Table 6:** Immediate adverse events reported in patients after HUCMSC graft infusion

Adverse event	Cases
Hyperthermia	0/225
Fever	5/225
Headache	1/225
Nausea	2/225
Vomiting	0/225
Erythema	0/225
Abdominal pain	0/225
Diarrhea	0/225
Hypertension/hypotension	0/225
Bradycardia/tachycardia	0/225

## Discussion

The quality control of CTPs is at least performed in three respects which include donor screening and starting material selection, in-process testing of cell products in GMP manufacturing, and release testing of the final product before clinic use [28]. In this study, we established a quality evaluation system to ensure the safety of therapeutic HUCMSCs as a CTP in clinic. We integrated various aspects regarding the characteristics and efficacy of HUCMSCs into a practical system of safety evaluation and quality control, which included GMP environmental monitoring program, quality control of critical raw materials and reagents, donor screening criteria and method, microbial contamination, cell viability, apoptosis, proliferation, cell cycle and growth curve, expressions of surface markers, differentiation potentials, karyotype analysis and tumorigenicity, and immune regulation capability and release assays.

The HUCMSC manufacturing facility and environment must meet the requirements of GMP for pharmaceutical manufacturers to minimize the particle and microorganisms in the final cell therapy product as far as possible [16]. It is relatively easy to design and construct a GMP-grade cell facility; the difficulty lies in implementing an effective environmental monitoring and maintenance program to smoothly run the cell bank. Environmental monitoring programs implemented in our center include physical environment, microbiological, and personnel controls. The environmental parameters of GMP laboratory such as lighting, temperature, humidity, air pressure, airflow volume, and velocity must be strictly controlled to ensure all parameters reach the GMP standards before processing. Acceptance criteria for temperature and relative humidity are 22 ± 2 °C and 30~50%, respectively. Environmental microbiological monitoring in the laboratory includes the detection of air and surface (laminar flow cabinet). The air plate sedimentation method was used for microbiological monitoring, and microbial contamination levels were expressed by counting cfu and particle monitoring; the alert and action limits for GMP laboratory and laminar flow cabinet are 4 cfu/30 min/dish (150 cfu/m^3^) and 0.2 cfu/30 min/dish (5 cfu/m^3^), respectively. Laboratory workers are the main source of microorganisms in the cell facility, and they can significantly affect the quality of product processing environment. To ensure that all personnel (even those who are in charge of cleaning and maintenance) receive appropriate training before entering the GMP lab, the training includes sterile operation, personal hygiene, laboratory contamination, and GMP-related knowledge. In addition to the relevant training, regular personal sampling monitoring was conducted too, such as gloves and work clothes before and after the operation and cultured with agar plates. After incubation, the results are expressed by counting cfu; the alert and action limits are 5 cfu/cm^2^ according to “Hygienic standard for disinfection in hospitals GB15982-2012” in China.

MSC donors must undergo strict eligibility determinations to screen healthy donors without transmit infectious diseases, malignant tumors, mental illness, and refractory chronic diseases. In our previous study, we found that even if HUCMSCs were derived from HBV women, the current cell-based testing assays failed to detect HBV in these cells, indicating that cell-level virus detection is not reliable in cell-based therapy [19]. Thus, we put forward that MSC donors should undergo other communicable disease tests at 3 months after the first serological communicable disease screening to exclude the potential window period of these viral infections in donors. In our practice, donors undergo communicable disease tests twice including HBV, HCV, HIV I and II, syphilis, CMV, and EBV, and in all tests, diseases must be negative in eligible donors. If the second serological screening is not available from the donor 3 months later, or any of the tested infectious diseases is positive in the second serological screening, the cultured cells from this donor are judged to be unqualified for clinic use based on the agreements in prior written consent, and all the expanded cells will be discarded as medical waste, even the cells have passed the systematic quality evaluation.

MSCs are broadly used in a variety of diseases due to their diverse physiological features such as preferential migration to damaged tissues, differentiation potentials of various cell types, and secreting tissue-renewing anti-inflammatory factors [29–32]. The parameters of HUCMSCs’ quality include identity, purity, viability, safety, and potency. Of which, the most challenging parameter is the potency test for MSC-based products because it should represent one or more of the cells’ relevant functions in vivo [28, 33]. Therefore, the potency assay of HUCMSCs should include not solely cellular phenotype assay, but also functionality relevant assay. In our quality system of HUCMSCs, we test the surface markers of HUCMSCs (positive CD73, CD105, and CD90; negative CD19, CD45, CD11b, CD34, and HLA-DR), viability, apoptosis, proliferation rate, growth curve, tumorigenicity, and karyotype analysis to assay identity, purity, viability, and safety of cells. In viability and growth assays, multiple methods are performed including trypan blue exclusion, apoptosis, EDU immunostaining, and cell cycle analysis. In addition, in these assays, a tumor cell line was used to act as a positive control because of the relatively stable characteristics of the tumor cell line. It also could be used as a reference standard to compare the viability and growth of different batches of HUCMSCs derived from different donors. For potency assay, we focused on the differentiation potentials into osteoblasts, adipocytes, and chondroblasts, and general immunomodulatory role of HUCMSCs. In differentiation assay, we established positive controls such as mouse adipose tissue section staining for adipocyte differentiation, mouse bone section staining for osteocyte differentiation, and mouse chondrocyte bone section staining for chondrocyte differentiation to evaluate whether the differentiation experiments are successful and the sufficiently potent or sub-sufficiently potent batches.

MSCs have been explored for a wide range of hyper-activated immune disorders such as transplant rejection, GVHD, and autoimmune diseases, due to the immunomodulatory activity of MSCs [34, 35]. The mechanism by which MSCs play the general immunomodulatory effects involves the orchestration of immune tolerance, then regulating the functions of regulatory B and T cells and innate suppressor cells as well [36]. In this study, we assayed T subpopulation of PBMCs by co-culturing with HUCMSCs to evaluate the immunomodulatory effects of HUCMSCs. HUCMSCs remarkably inhibited the proliferation of CFSE-labeled PBMCs by co-culturing and suppressed the activation and differentiations of CD4+ T cells into Th1 and Th17 subpopulations. In addition, HUCMSCs exert the maturation of Treg subpopulation in PBMCs induced by IL-2. Overall, MSCs could skew the inflammatory niche into an anti-inflammatory one, via direct and indirect immunoregulatory activities of Tregs and monocytes [37, 38].

Before being released for clinical application, each HUCMSC batch must undergo release testing assay. Because the HUCMSCs have undergone a systemic in-processing quality assay and were judged to be a qualified cell product, the release assay should be relatively simple and be finished quickly. In our system, the release assay includes bacterial, fungi, endotoxin, mycoplasma, cell amount, and viability as the final safety assurance for recipients. The qualified HUCMSCs adjudged based on the results after the in-process and release assessments were tested for treating a variety of diseases including premature ovarian failure (POF), coronary artery disease (CAD), diabetic erectile dysfunction, type 1 diabetes mellitus, nasal septum injury, and liver failure in our hospital. In total, 225 patients received HUCMSC treatment, only 5 cases had a transient slight fever, one case had a headache, and 2 cases had nausea within 2 days after HUCMSC transplant; no other adverse outcomes were observed during the 1-year follow-up period, indicating our established systemic quality control and assessment were effective to ensure the quality and safety of HUCMSCs as cell therapy products in clinical application.

As outlined earlier in our study, our system only established a minimum set of standards for HUCMSCs, and there were some shortcomings due to the limited understanding of the MSC mechanism of action in stem cell therapy. Each MSC product has unique biological characteristics due to tissue origin, donor conditions (age, gender, and individual heritability), passage number, cell preparation, and pre-modulation before application. Thus, each MSC product requires definitive criteria in every potent assay such as differentiation potential and immune regulation according to its own characteristics. In addition, although HUCMSCs have been successfully tested for various indications in the pre-clinical phase or clinical trials, its mechanism differs among indications. For example, in the field of immunotherapy, the immunomodulatory actions of HUCMSCs are considered to exert its therapeutic effects, but not the differentiation potential. Thus, some potent assays regarding immune regulations are preferred above other assays such as adipogenic, chondrogenic, and osteogenic differentiation potentials. Therefore, it needs to develop specific potent assays in line with a certain indication with a further understanding of MSCs and diseases.

## Conclusion

In this study, we establish a systemic quality control and potent assays to guarantee the safety and effectiveness of HUCMSCs based on a minimum set of standards in MSC-based product. The qualified HUCMSCs were tested for various indications, and no severe adverse reaction was observed during the 1-year follow-up period, indicating our system is valid for quality control and assessment of HUCMSCs as a cell-based product.

## Supplementary information

**Additional file 1 **: **Table S1.** The list of test bovine-specific viruses and corresponding antibodies.

**Additional file 2 **: **Figure S1.** The viability of HUCMSCs suspended in saline with or without 5% HSA. The viability of HUCMSCs suspended in saline with 5% HSA was more than 90% within 8 hours, with a much better viability than HUCMSCs in alone saline solution at all indicated time points.( *, *p* < 0.05; **, *p* < 0.01; ***, *p* < 0.001).

## Data Availability

The datasets used and/or analyzed during the current study are available from the corresponding author on reasonable request.

## Abbreviations

MSCs: Mesenchymal stromal cells
CTP: Cell-based therapy product
GVHD: Graft-versus-host disease
HUCMSCs: Human umbilical cord mesenchymal stromal cells
MCB: Master Cell Bank
WCB: Working Cell Bank
GMP: Good Manufacturing Practice
FBS: Fetal bovine serum
PBS: Phosphate-buffered saline
BVDV: Bovine viral diarrhea virus
BPIV: Bovine parainfluenza virus
BPV: Bovine parvovirus
BAV: Bovine adenovirus
REO: Reovirus
BT: Bovine turbinate
HBV: Hepatitis B virus
HCV: Hepatitis C virus
HIV: Human immunodeficiency virus
CMV: Cytomegalovirus
EBV: Epstein-Barr virus
ISCT: International Society for Cellular Therapy
FITC: Fluroisothiocyanate
PE: Phycoerythrin
SCID: Severe combined immune deficiency
HESCs: Human embryonic stem cells
H&E: Hematoxylin-eosin
SLE: Systemic lupus erythematous
PBMCs: Peripheral blood mononuclear cells
POF: Premature ovarian failure
CAD: Coronary artery disease
SD: Standard deviation
