# Supplementary material for: The quality evaluation system establishment of mesenchymal stromal cells for cell-based therapy products
Source: Stem Cell Res Ther. 2020 May 13;11:176. doi: 10.1186/s13287-020-01696-6 (PMC7222464; doi:10.1186/s13287-020-01696-6)
Supplement: Supplementary file 1 — Additional file 1 : Table S1. The list of test bovine-specific viruses and corresponding antibodies. [file 13287_2020_1696_MOESM1_ESM.docx]

Supplementary Table 1. The list of test bovine-specific viruses and corresponding antibodies

| Bovine viruses | Antibodies (Catalogue NO., Company) | Release Criteria |
| --- | --- | --- |
| Bovine parainfluenza virus (BPIV) | Immunofluorescence (CJ-F-PI3-10 ML,VMRD) | Negative |
| Bovine adenovirus (BAV) | Immunofluorescence (CJ-F-BAV-10 ML,VMRD) | Negative |
| Bovine Parvo virus (BPV) | Immunofluorescence (CJ-F-BPV-10 ML,VMRD) | Negative |
| Bovine viral diarrhea virus (BVDV) | Immunofluorescence (BA2,VMRD) | Negative |
| Reovirus (REO) | Immunofluorescence (CJ-F-REO-10 ML,VMRD) | Negative |
